# Thrombotic Thrombocytopenic Purpura and Metastatic Malignancy-Like Presentation Secondary to Hypervirulent Strain of Klebsiella pneumoniae: A Case Series

**DOI:** 10.7759/cureus.28209

**Published:** 2022-08-20

**Authors:** Mohamad Bakir, Fatima Rebh, Mohammad A Khan

**Affiliations:** 1 College of Medicine, Alfaisal University, Riyadh, SAU; 2 Department of Internal Medicine, Section of Infectious Diseases, Prince Mohammed Bin Abdulaziz Hospital, Riyadh, SAU; 3 Laboratory and Blood Bank Department, Microbiology Laboratory, Prince Mohammed Bin Abdulaziz Hospital, Riyadh, SAU

**Keywords:** hypervirulent klebsiella pneumoniae, klebsiella pneumoniae invasive syndrome, klebsiella pneumoniae, case report, hypermucoviscous, hypervirulent strain, complication, hematologic emergency, thrombotic thrombocytopenic purpura

## Abstract

Thrombotic thrombocytopenic purpura (TTP) is a rare and potentially fatal disease. The majority of cases are caused by a significant enzyme deficiency in the blood called the von Willebrand factor (VWF) cleaving protease (also called ADAMTS13). TTP is classified as a hematologic emergency because of the high mortality rate. The diagnosis is difficult due to the extensive overlap in the clinical manifestations of TTP and other illnesses. Klebsiella pneumoniae infection can in very rare instances present with TTP and/or a metastatic-like presentation where the patient might have prostate, liver, brain, and lung abscesses mimicking late-stage solid organ malignancy.

In this paper, we report a case of TTP secondary to *Klebsiella pneumoniae* infection in a 38-year-old male patient, who presented with fever, cough, and shortness of breath for five days. On examination, he was vitally unstable, confused, and not oriented, with a Glasgow Coma Scale (GCS) of 9/15. Complete blood count (CBC) showed a high white blood cell (WBC) count, very low platelet count, increased reticulocyte count, and significant elevation of schistocytes on peripheral blood film. Sputum and blood cultures were positive for *Klebsiella pneumoniae*. Computerized tomography (CT) scan chest showed bilateral lung parenchymal nodules. An abdominal ultrasound (US) scan detected a right hepatic lobe lesion that was both cystic and solid. The patient was initially started on meropenem, vancomycin, and levofloxacin due to shock presentation which was de-escalated to ceftriaxone later. The patient had five therapeutic plasma exchange sessions and was started on methylprednisolone for three days. The patient's situation gradually improved, and he was discharged later on.

The second case is a 63-year-old-male patient who presented with fever, dry cough, night sweats, and dysuria for seven days. He was vitally stable, conscious, alert, and oriented. His hemoglobin was 9.6 g/dl. He was scheduled for an urgent colonoscopy to rule out colon cancer along with computed tomography (CT) scan of the chest, abdomen, and pelvis. The CT scan showed complex cystic lesions involving the right hepatic lobe, lungs, adrenal glands, and prostate. The clinical picture was suggestive of hyper-mucoid *Klebsiella pneumoniae* infection showering to the liver, adrenal glands, and prostate. A drained prostate collection and urine cultures confirmed the diagnosis. The patient was managed with surgical drainage of the collection in addition to ceftriaxone and metronidazole. The patient was discharged in good health on ciprofloxacin with follow-up as an outpatient.

## Introduction

Thrombotic thrombocytopenic purpura (TTP) is a rare disorder with an instance of two to six per million population. TTP is caused by autoantibodies to the ADAMTS13 protease (a disintegrin and metalloproteinase with thrombospondin type one motifs 13). Normally, the high-molecular-weight VWF (Von Willebrand Factor) multimers are cleaved into smaller, less functional molecules by ADAMTS13 protease. In severe ADAMTS13 deficiency (activity of less than 10% of normal), ultra-large VWF multimers accumulate and aggregate with platelets, resulting in occlusive microvascular platelet thrombi [1-3]. Thrombocytopenia, microangiopathic hemolytic anemia (MAHA), fever, neurologic symptoms, and renal impairment are the classic pentad of TTP presentations, though not all findings are present in every patient [4]. Accurate and quick detection and treatment of the illness is crucial due to the high mortality rate that reaches up to 90% of untreated patients [3]. Elevated indirect bilirubin, decreased to absent haptoglobin, increased lactate dehydrogenase, hemosiderinuria, hemoglobinuria, and the presence of schistocytes on the peripheral blood smear, support initial suspicion of TTP [5]. In TTP, the platelet count is markedly reduced, with platelet counts of less than 20 x 10^9^/L; however, the symptoms are quite general and can be seen in various medical disorders such as hemolytic uremic syndrome (HUS) and disseminated intravascular coagulation (DIC) [1,2]. *Klebsiella pneumoniae* is a pathogen well known for its relationship with invasive liver abscess syndrome (ILAS), a complicated infection [6]. ILAS is linked to pyogenic liver abscesses and various infection sites throughout the body [7]. ILAS develops as a result of a hypervirulent strain of *Klebsiella pneumoniae* that generates a hyper capsule. Hypermucoviscosity (HMV) phenotype is a known virulent factor of *Klebsiella pneumoniae*, which can be confirmed by a string test in the microbiology lab. If a viscous string longer than 5 mm is created when a loop is used to stretch the colony on an agar plate, the string test is considered positive [8]. There is an enhanced generation of polysaccharides in this strain of *Klebsiella pneumoniae*, which makes it resistant to usual immune responses, such as complement activation and phagocytosis [9]. As a result, the illness, which is frequently acquired in the community, can affect both immunocompetent and immunocompromised patients. In our case report, we are presenting two rare presentations of Klebsiella infection with positive string test in both, the first case presented with TTP, and the second case presented with fever, anemia, and a CT scan finding of disseminated collections mimicking metastatic malignancy. These cases demonstrate the complex presentation of the hypervirulent strain of Klebsiella infection. 

## Case presentation

Case 1 

**Figure 1:**
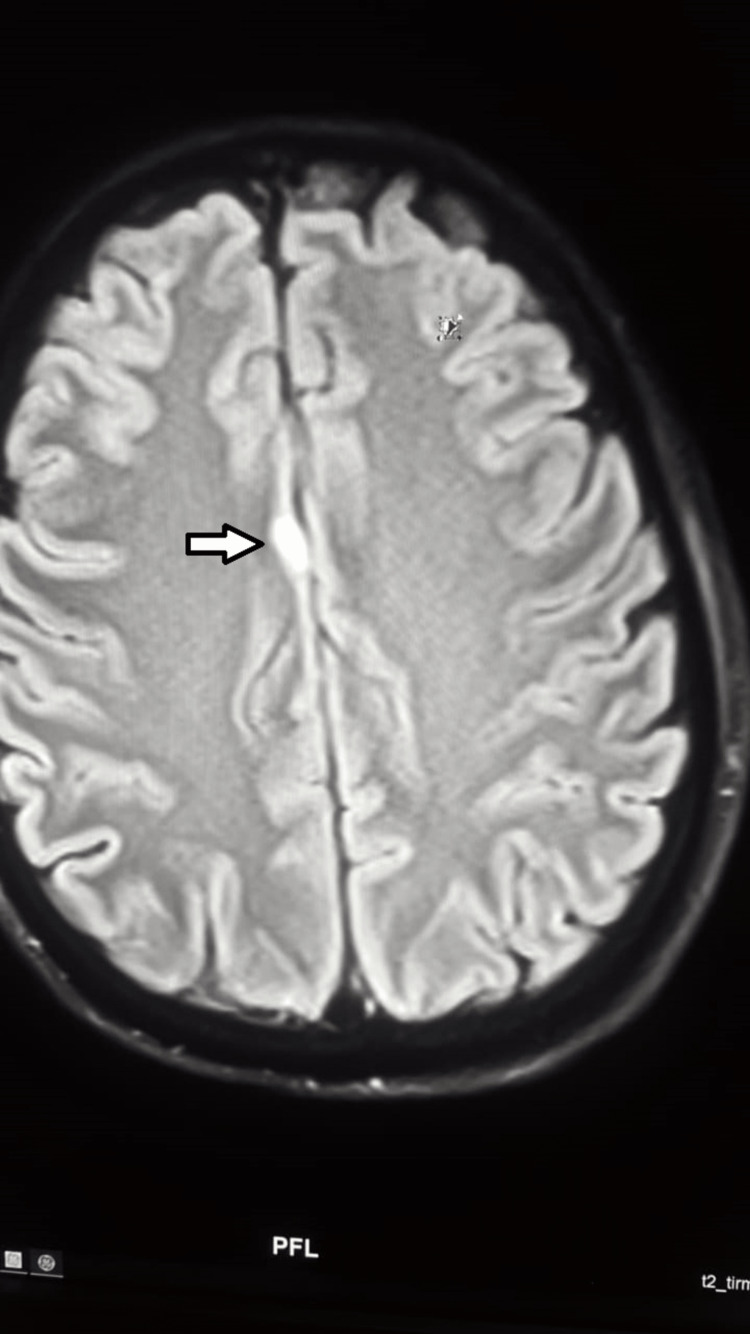
A cortical fluid-attenuated inversion recovery (FLAIR) image A hyperintense lesion in the medial side of the right frontal lobe with restricted diffusion in the diffusion-weighted imaging (DWI), representing a small acute infarction (white arrow).

**Figure 2:**
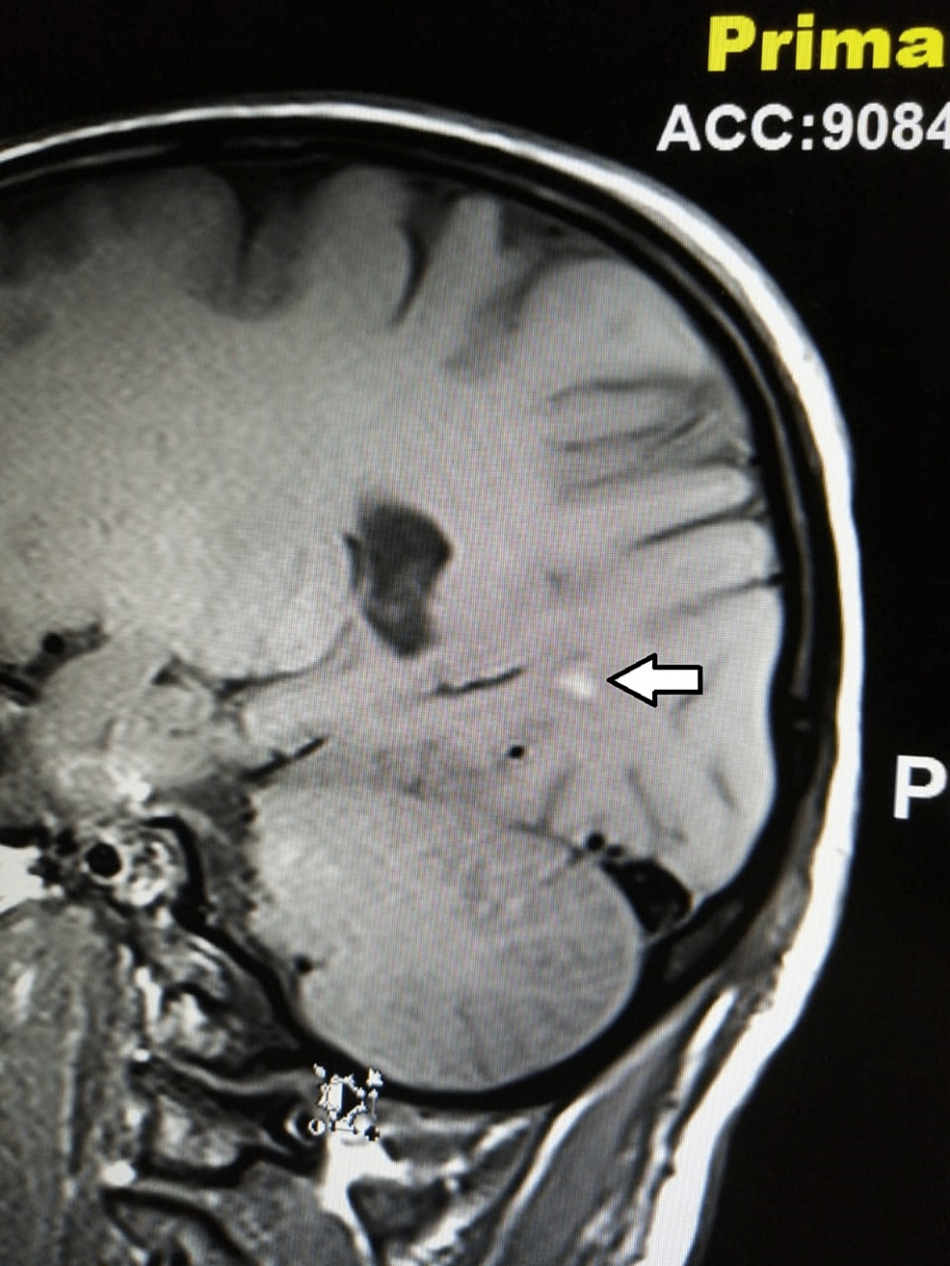
T1-weighted MRI image The image shows a hyperintense focus in the white matter of the right occipital lobe, representing a tiny hemorrhage (white arrow).

A 38-year-old male patient, known to have diabetes mellitus type 2, presented to the emergency department on July 1st, 2020, with fever, cough, and shortness of breath for five days. On examination, he was vitally unstable, with a respiratory rate of 50 breaths per minute, oxygen saturation of 91% on 15 liters non-breathable mask, and a mean arterial pressure of 80. He was confused and not oriented to time, place, or person, with a GCS of 9/15. Therefore, he was admitted to the intensive care unit on mechanical ventilation for 12 days. After completion of 17 days in the ICU, he was shifted to the general ward. On admission, a complete blood count (CBC) showed a high WBC count of 20000 cells per microliter, a very low platelet count of 6 x 10^9^/L, an increased reticulocyte count of 11%, and schistocytes were present on the blood film. In addition, bilirubin and international normalized ratio (INR) were both elevated. Sputum and blood cultures were obtained on admission, and they were positive for *Klebsiella pneumoniae*. The patient was started on empirical antibiotic therapy. Since lumbar puncture could not be done due to low platelet count, a CT scan brain was done to rule out meningitis, and it was unremarkable. However, due to the patient's continuing confusion and poor improvement in the level of consciousness, magnetic resonance imaging (MRI) was obtained, and a small 1 cm area of the cortical hyperintense signal was detected at the mesial gyrus adjacent to the corpus callosum. This 1 cm area shows restricted diffusion and could represent an inflammatory process such as encephalitis. In addition, the MRI showed hyperintense focus in the white matter of the right occipital lobe, indicating a microhemorrhage (Figures 1, 2).

**Figure 3:**
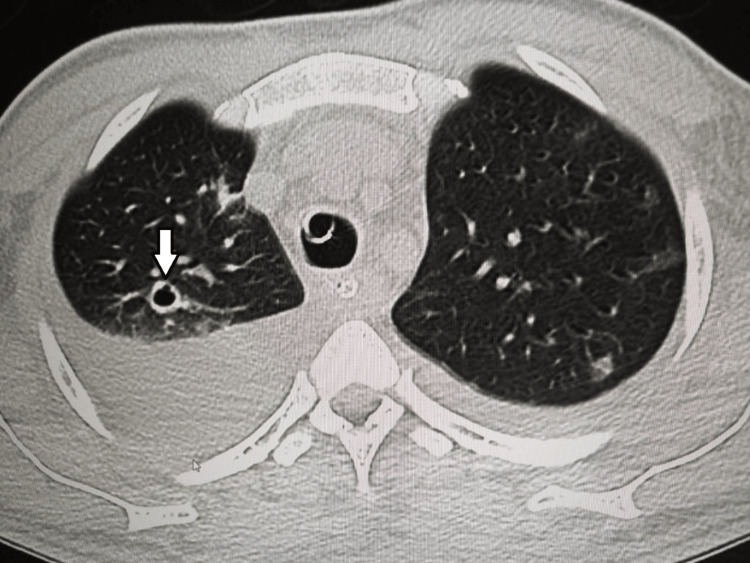
A CT scan of the chest The scan shows a right upper lobe cavitary nodule (white arrow) with left lung ground-glass nodules and bilateral pleural effusion.

**Figure 4:**
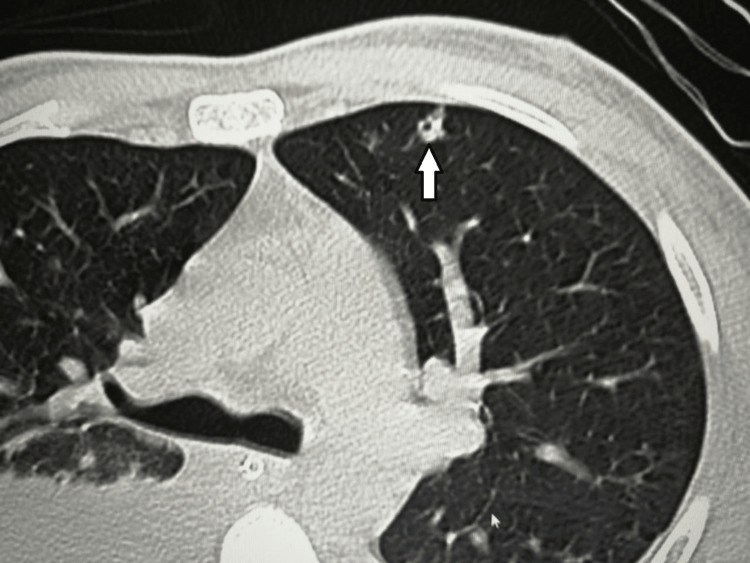
A CT scan of the chest The scan shows a small cavitary nodule in the anterior segment of the left upper lobe (white arrow).

Even though the patient was started on empirical antibiotics, his confusion did not improve, so viral meningitis had to be ruled out. A lumbar puncture was conducted after the improvement of the patient’s platelet count. CSF showed a WBC count of 25 cells/mm^3^, mainly lymphocytes, a protein count of 836 mg/dl, and a glucose level of 7.39 mmol/L. Since the CSF collection was obtained after antimicrobial initiation, the CSF fluid culture reported no growth, and a molecular test for viral meningitis was sent for HSV PCR (herpes simplex virus polymerase chain reaction) and came back negative. The CT scan of the chest showed bilateral lung parenchymal variable-sized, unevenly distributed nodules. Part of them was showing cavitary changes, and the picture was suggestive of septic emboli (Figures 3, 4).

**Figure 5:**
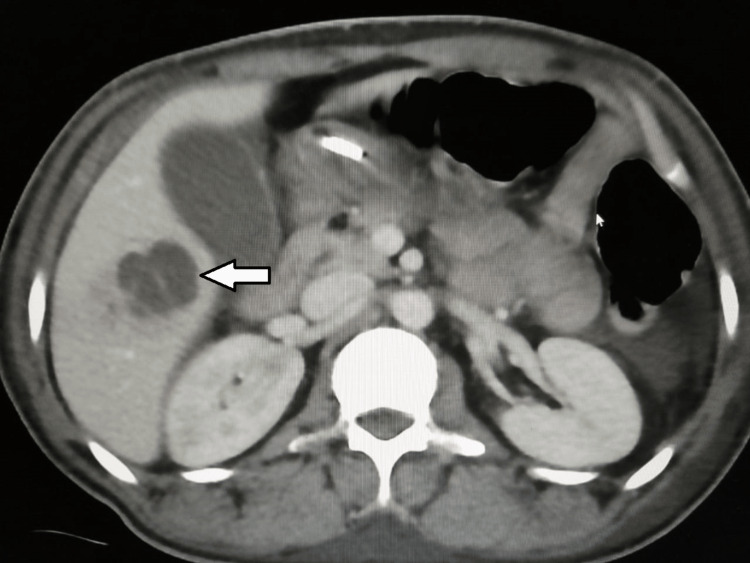
Abdominal CT scan The scan shows a septated cystic lesion in segment 5/6 of the liver (white arrow).

**Figure 6:**
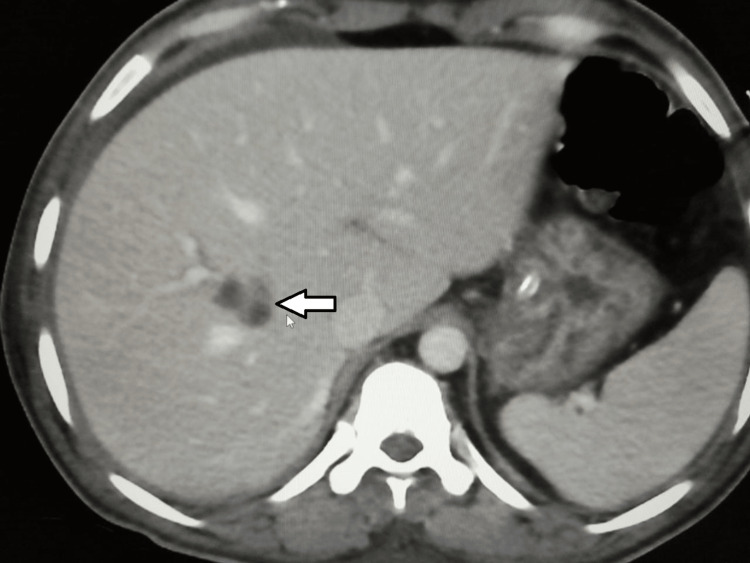
Abdominal CT scan The scan shows a small septated cystic lesion in segment 8/1 of the liver (white arrow).

Further, an abdominal CT scan showed a rounded hypodense hepatic lesion in segment 5, seen with small internal septations, measuring about 3 x 3.2 cm with an adjacent ill-defined hepatic parenchymal faint hypodense area, likely related to infectious etiology (Figure 5). There was another small lobulated hypodense hepatic lesion seen in segment 8 measuring about 2.4 x 1.4 cm (Figure 6). 

**Figure 7:**
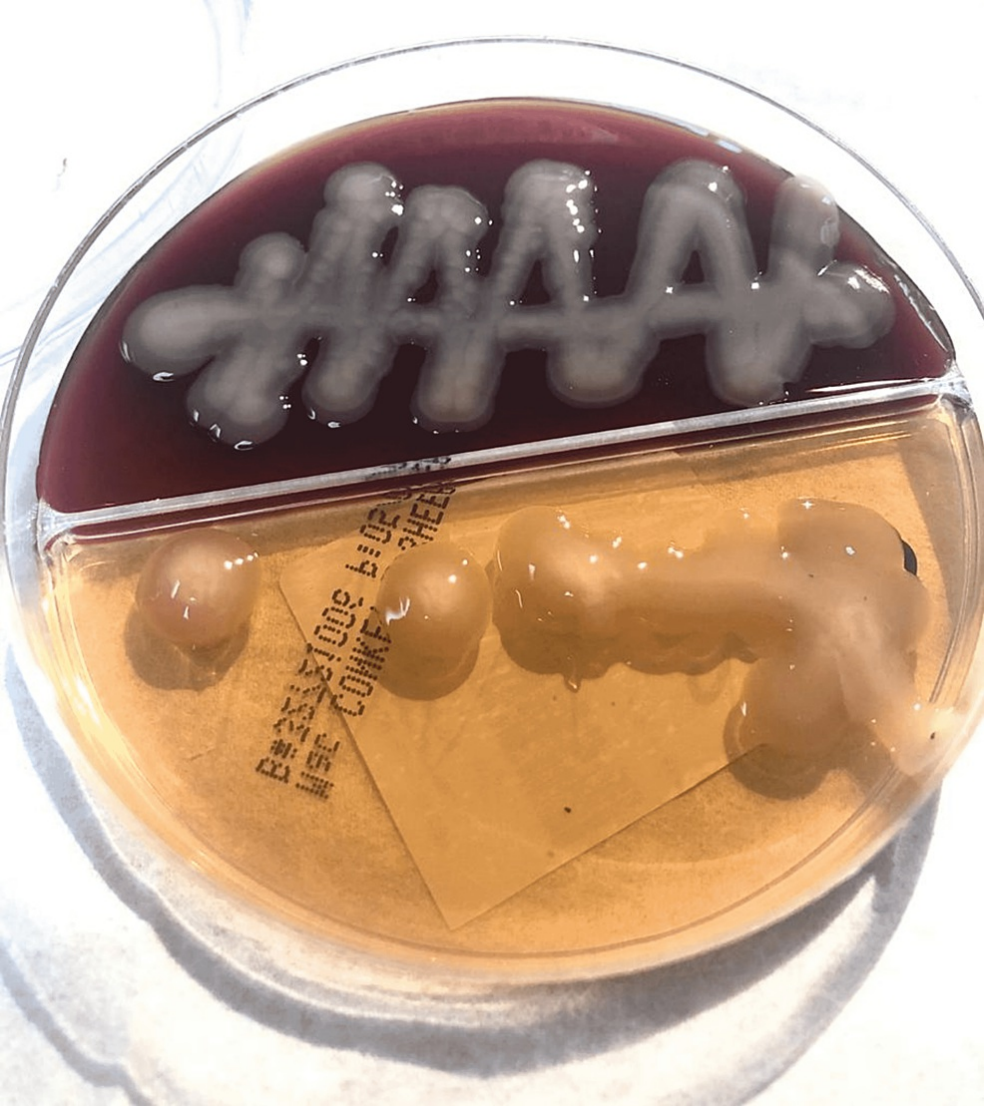
MacConkey agar *Klebsiella pneumoniae* growth on MacConkey agar with smooth, elevated mucoid colonies.

**Figure 8:**
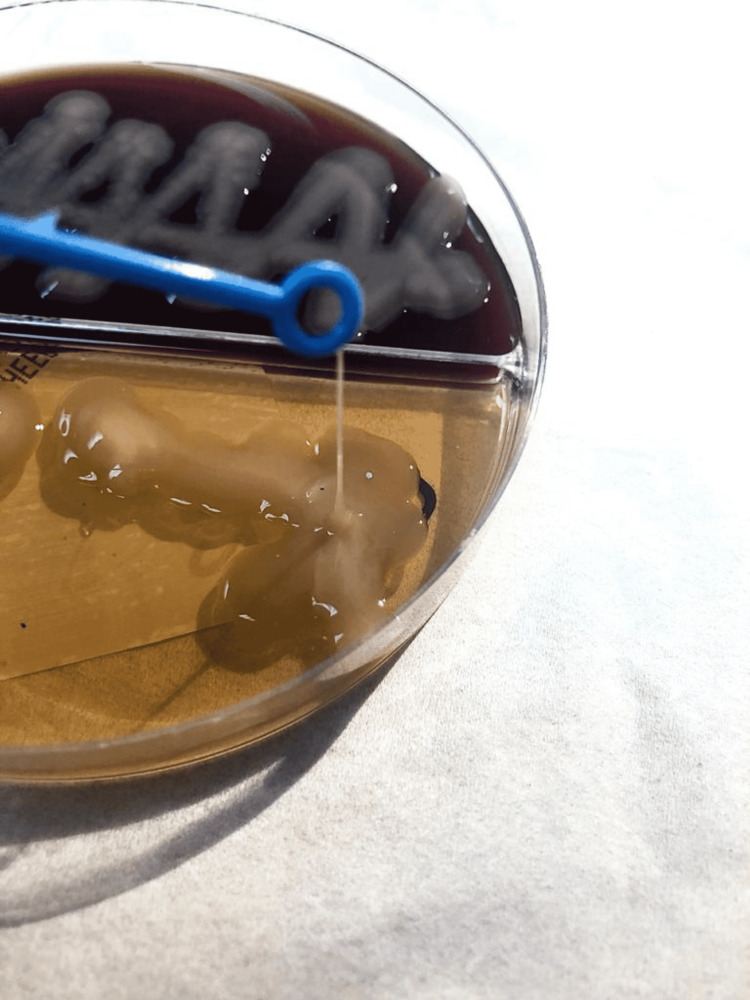
A positive string test String length greater than 5mm shows a positive string test and the hypermucoviscous type.

Sputum, peritoneal fluid, and blood cultures were collected, and Klebsiella pneumoniae was isolated from all specimens. The string test was positive (Figures 7, 8). We confirmed the identification of *Klebsiella pneumoniae* infection with the Vitek 2 automated identification instrument (bioMérieux SA, Marcy-l'Étoile, France).

The diagnosis of disseminated *Klebsiella pneumoniae* infection (blood, lungs, peritoneum, liver, and brain) was established. The patient was started on meropenem, vancomycin, and levofloxacin initially due to clinical instability, then switched to ceftriaxone for six weeks, guided by the susceptibility culture report, in addition to the drainage of the liver abscess as a source control management. A diagnosis of TTP was made after a full hematology workup. The patient had five therapeutic plasma exchange sessions and was started on methylprednisolone for three days. The patient was discharged in stable conditions. 

Case 2 

**Figure 9:**
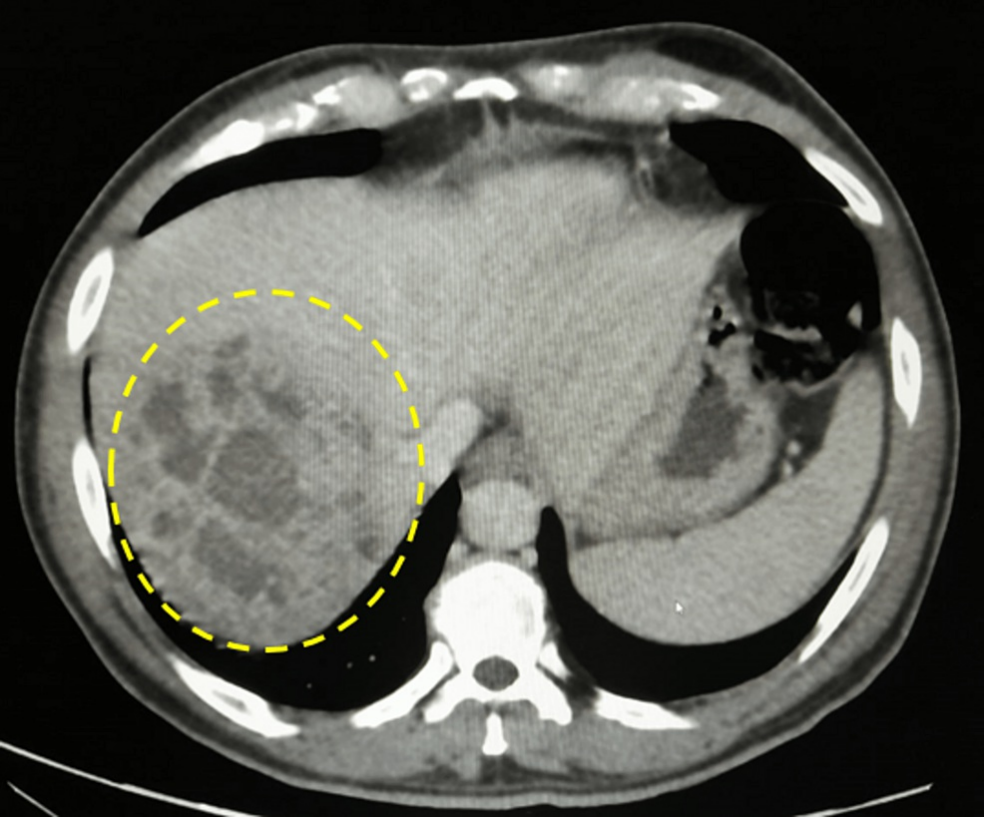
Abdominal CT scan The scan shows large multiloculated cystic lesions in segment 8/7 of the liver (dashed circle).

A 63-year-old male patient, known case of type 2 diabetes mellitus, hypertension, and internal hemorrhoids with recurrent painless rectal bleeding of fresh blood for three months, presented with fever of 38 °C, dry cough, and dysuria for seven days before admission, in addition to night sweats and weight loss for the last four months. On examination, the patient was fatigued and pale in appearance; his vital signs were stable, and he was conscious, alert, and oriented. The abdomen was soft and lax with no tenderness or masses. Rectal examination showed no external piles, fissures, or masses, however, an anoscope showed internal piles at the 3 o’clock location with engorged veins. His hemoglobin was 9.6 g/dl. Accordingly, he was scheduled for an urgent colonoscopy along with a CT scan of his abdomen and pelvis to rule out malignancy. A CT scan of the abdomen and pelvis with intravenous (IV) contrast was performed in the portal venous phase. No gross colonic masses were identified; however, the liver demonstrated an ill-defined heterogeneous multiloculated complex cystic lesion involving the right hepatic lobe, measuring collectively about 10 x 10 cm, likely representing abscess formation (Figure 9).

**Figure 10:**
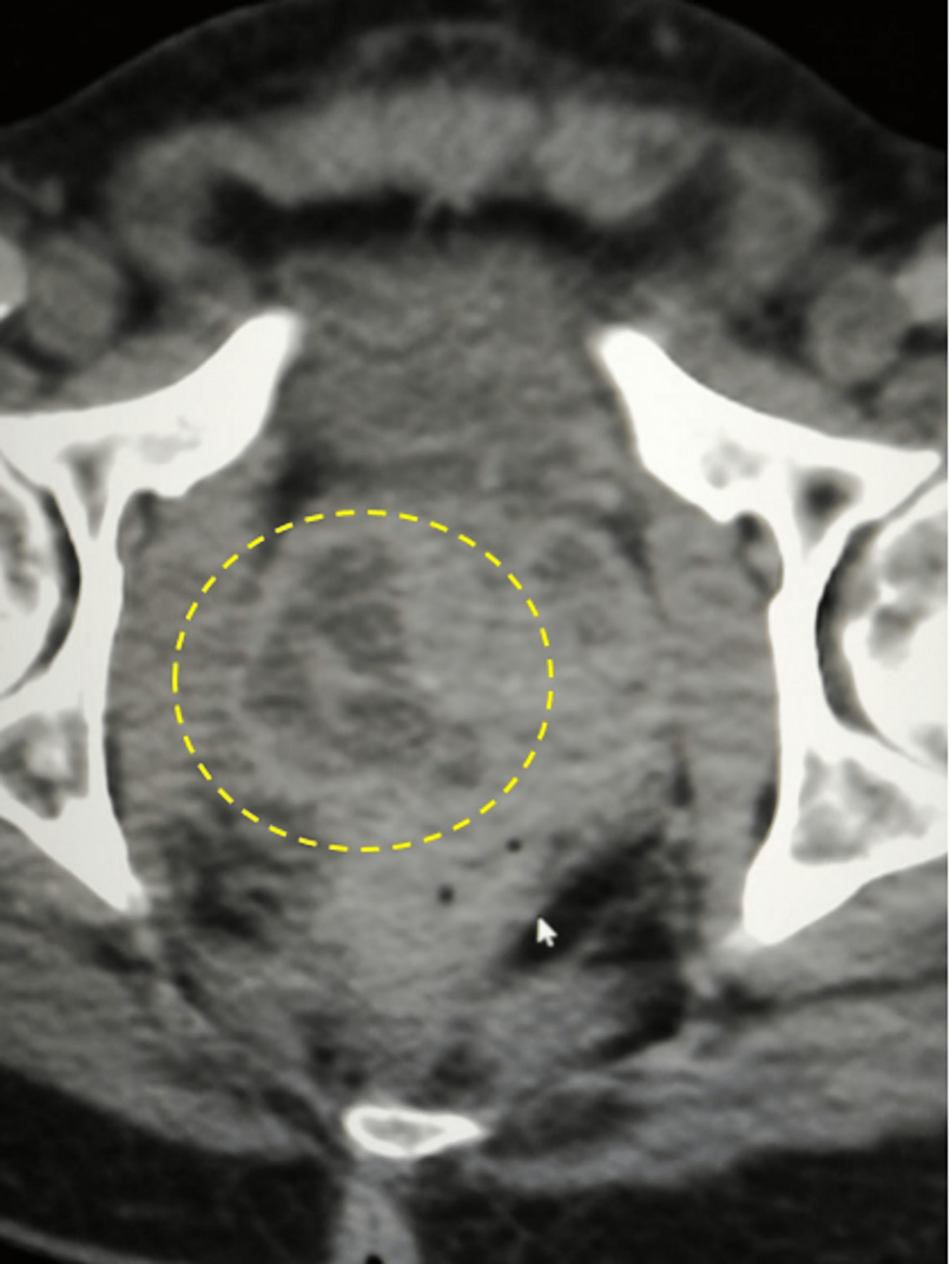
Pelvic CT scan The scan shows loculated prostatic abscesses (dashed circle).

There were large heterogeneous multiloculated cystic lesions seen, involving the prostate, mainly on the right side, measuring 4.4 x 4.3 x 4 cm with surrounding peripheral enhancement and surrounding stranding (Figure 10).

**Figure 11:**
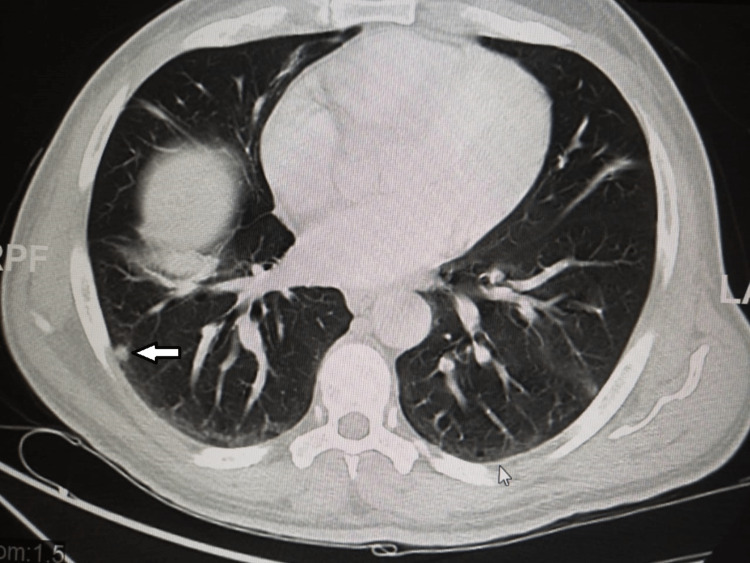
A CT scan of the chest The scan shows a small sub-pleural nodule-like consolidation (white arrow).

**Figure 12:**
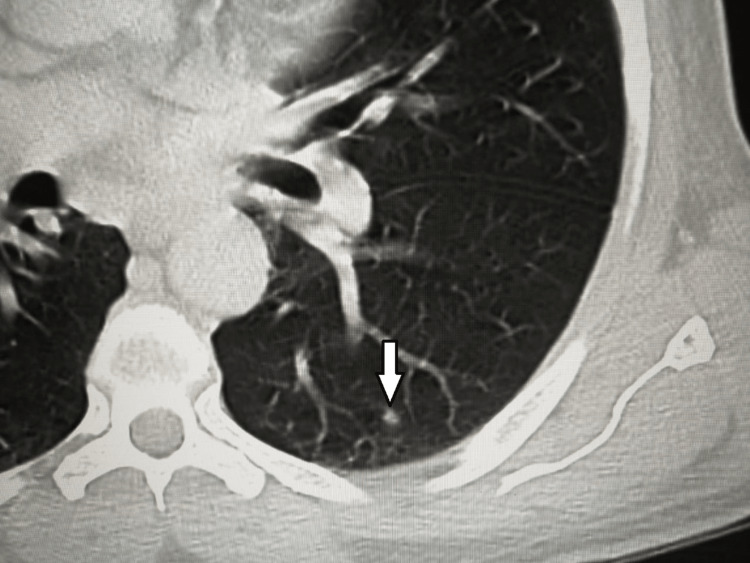
A CT scan of the chest The scan shows a small nodule-like consolidation in the lower lobe of the left lung (white arrow).

Moreover, the CT chest with contrast showed multiple scattered small pulmonary nodules, seen in the left upper and bilateral lower lung lobes, with the largest seen in the right lower lobe measuring 0.8 x 0.5 cm, likely representing pulmonary metastasis (Figures 11, 12).

**Figure 13:**
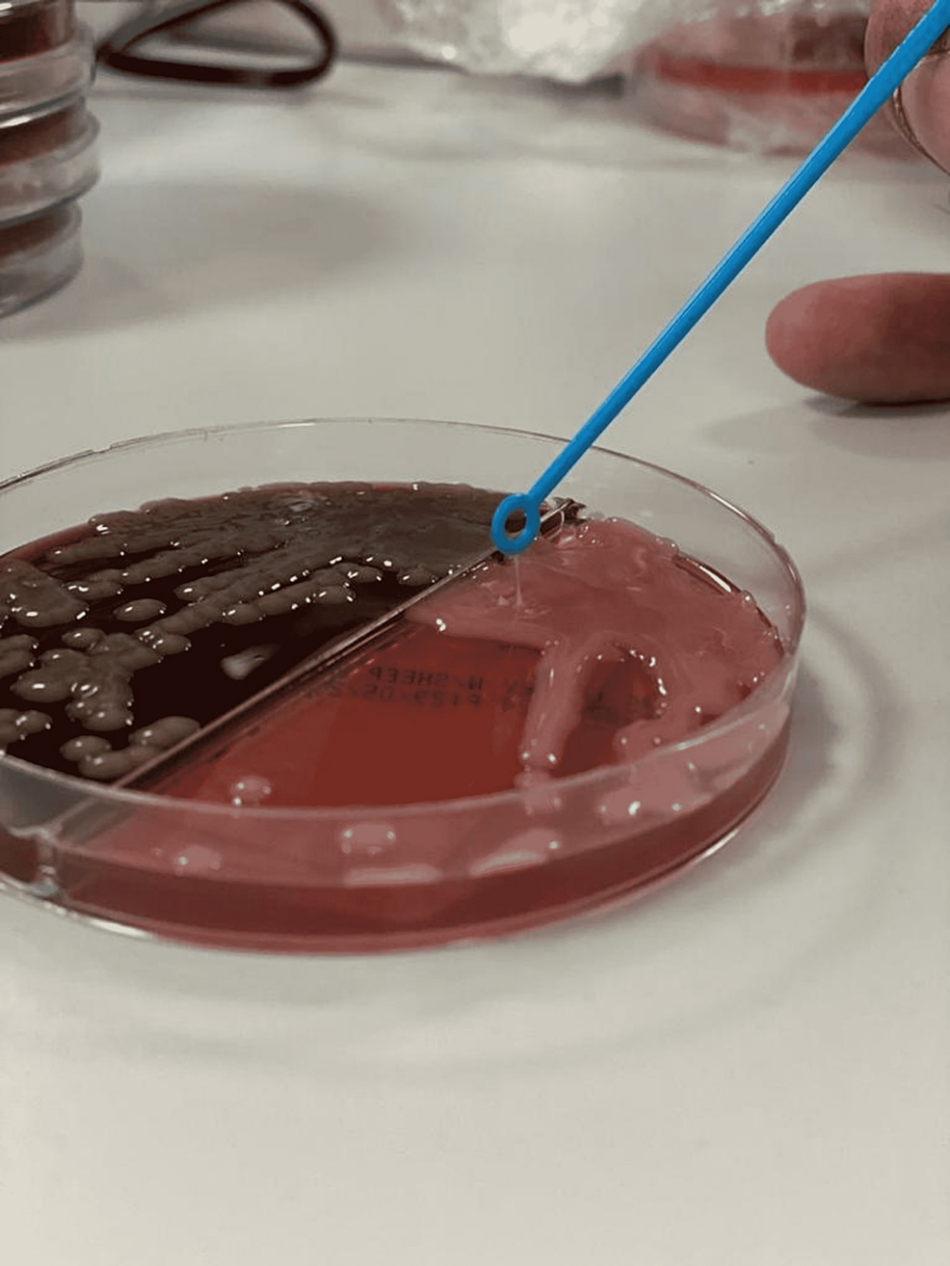
Klebsiella pneumoniae growth on MacConkey agar The MacConkey agar shows smooth, elevated mucoid colonies, with a positive string test.

Transthoracic echocardiogram was normal. The surgical pathology of the prostate tissue revealed benign prostatic hyperplasia associated with acute prostatitis, with no evidence of malignant cells. A urine sample and a collection of prostate fluid were drained and sent for culture. *Klebsiella pneumoniae* was isolated from both specimens with a positive string test (Figure 13) (Video 1). We confirmed the identification of *Klebsiella pneumoniae* with the Vitek2 automated identification instrument.

**Video 1:** A positive string test formed when touched with a loop, illustrating the hypermucoviscous phenotype.

Therefore, the evidence of disseminated hypermucoid *Klebsiella pneumoniae* infection showering to the prostate, liver, and lungs was confirmed. The patient was managed with surgical drainage of the collection, in addition to ceftriaxone. The patient was discharged in good health on ciprofloxacin with follow-up as an outpatient. 

## Discussion

*Klebsiella pneumoniae* is a gram-negative, enveloped, non-motile bacterium belonging to the Enterobacteriaceae family. This microorganism is present in the environment and has been linked to pneumonia in patients with diabetes mellitus or alcohol use disorder [10]. *Klebsiella pneumoniae* commonly colonizes human mucosal surfaces of the oropharynx and the gastrointestinal (GI) tract, and once within the body, the bacterium can exhibit significant levels of virulence and drug resistance [10]. The bacteria's polysaccharide capsule is the most essential virulence component, allowing them to avoid opsonophagocytosis and serum death by the host organism.

Lipopolysaccharides, which cover the outer surface of gram-negative bacteria, are a second virulence factor [10]. Reports from Taiwan in the mid-1980s and 1990s described a distinct clinical presentation of community-acquired *Klebsiella pneumoniae* infections. Patients with no history of hepatobiliary disease appeared with community-acquired pyogenic liver abscesses (CA-PLA) and a tendency for distant metastasis [11,12]. Colonies produced on an agar plate have a hypermucoviscous appearance. A positive "string test" defined this phenotype. The string test is affirmative when a bacteriology inoculation loop or needle can stretch bacterial colonies on an agar plate and form a viscous string longer than 5 mm in length [8]. Diabetes is one of the most well-defined risk factors for the hypervirulent strain of *Klebsiella pneumoniae* infection [13]. Poor glycemic management is thought to prevent adequate neutrophil phagocytosis of the hypervirulent capsular strain. Strict glycemic regulation has been demonstrated to lower the occurrence of metastatic infections in people with diabetes mellitus [14]. ILAS is misleading since it frequently appears as a metastatic infection affecting the liver, central nervous system, eye, musculoskeletal system, urinary tract, and lungs, among other places [6,13]. TTP is a rare blood condition characterized by clotting in small blood arteries (thromboses), resulting in a low platelet count. TTP in its full-blown form consists of the following pentad: microangiopathic hemolytic anemia, neurologic abnormalities, purpura, kidney disease, and fever [5]. In terms of the association between TTP, ADAMTS13, and infectious disorders, it has been observed that in sepsis patients, ADAMTS13 activity is reduced [15]. This could be explained in part by the inflammatory cytokine interleukin-6, which significantly inhibits ADAMTS13 activity, resulting in a slower rate of cleavage of ultra-large VWF multimers [16]. Most antibiotics have traditionally been effective against ILAS isolates. Third-generation cephalosporins remain the cornerstone of treatment because of their established penetration into the CSF fluid [17]. Ampicillin-sulbactam, aztreonam, and fluoroquinolones have also been used successfully. Extended-spectrum beta-lactamase-producing *Klebsiella pneumoniae* is still uncommon in ILAS, but when it does occur, it is usually treated with a carbapenem [6]. There is no formal recommendation for the duration of treatment. The duration of intra-abdominal infection is dependent on good source control, according to the Infectious Disease Society of America (IDSA) guidelines [18]. Overall, ILAS is routinely treated for four to six weeks, with some cases lasting longer depending on imaging. According to prior research, the average length of stay is roughly 23.3 days [13].

## Conclusions

TTP is a rare and life-threatening condition. The clinical features of TTP and other disorders sometimes overlap, making the diagnosis challenging. TTP can be, in very rare instances, secondary to *Klebsiella pneumoniae* infection. *Klebsiella pneumoniae* infection can also manifest as a metastatic-like presentation in which the patient has abscesses in different organs of his body such as the prostate, liver, brain, and lungs, similar to late-stage solid organ malignancy. The hypervirulent strain of *Klebsiella pneumoniae* is still susceptible to ceftriaxone in the majority of cases, and despite the severe presentation of this organism, the use of a narrower spectrum of cephalosporin is advised. Future studies are needed to confirm the link between *Klebsiella pneumoniae* infection, TTP, and metastatic-like presentation.
